# Endometrium metabolomic profiling reveals potential biomarkers for diagnosis of endometriosis at minimal-mild stages

**DOI:** 10.1186/s12958-018-0360-z

**Published:** 2018-04-30

**Authors:** Jingjie Li, Lihuan Guan, Huizhen Zhang, Yue Gao, Jiahong Sun, Xiao Gong, Dongshun Li, Pan Chen, Xiaoyan Liang, Min Huang, Huichang Bi

**Affiliations:** 1 Center of Reproductive Medicine, the Sixth Affiliated Hospital, Sun Yat-sen University, Guangzhou, China; 2 School of Pharmaceutical Sciences in Sun Yat-sen University, 132# Waihuandong Road, Guangzhou, University City, Guangzhou, 510006 People’s Republic of China; 3 Pharmacy Department, the First Affiliated Hospital, Sun Yat-sen University, Guangzhou, China; 4 School of Public Health, Guangdong Pharmaceutical University, Guangzhou, China

**Keywords:** Endometriosis, Metabolomics, UHPLC-ESI-HRMS, Eutopic endometrium

## Abstract

**Background:**

The sensitivity and specificity of non-invasive diagnostic methods for endometriosis, especially at early stages, are not optimal. The clinical diagnostic indicator cancer antigen 125 (CA125) performs poorly in the diagnosis of minimal endometriosis, with a sensitivity of 24%. Therefore, it is urgent to explore novel diagnostic biomarkers. We evaluated the metabolomic profile variation of the eutopic endometrium between minimal-mild endometriosis patients and healthy women by ultra-high-performance liquid chromatography coupled with electrospray ionization high-resolution mass spectrometry (UHPLC-ESI-HRMS).

**Methods:**

Our study comprised 29 patients with laparoscopically confirmed endometriosis at stages I-II and 37 infertile women who underwent diagnostic laparoscopy combined with hysteroscopy from January 2014 to January 2015. Eutopic endometrium samples were collected by pipelle endometrial biopsy. The metabolites were quantified by UHPLC-ESI-HRMS. The best combination of biomarkers was then selected by performing step-wise logistic regression analysis with backward elimination.

**Results:**

Twelve metabolites were identified as endometriosis-associated biomarkers. The eutopic endometrium metabolomic profile of the endometriosis patients was characterized by a significant increase in the concentration of hypoxanthine, L-arginine, L-tyrosine, leucine, lysine, inosine, omega-3 arachidonic acid, guanosine, xanthosine, lysophosphatidylethanolamine and asparagine. In contrast, the concentration of uric acid was decreased. Metabolites were filtered by step-wise logistic regression with backward elimination, and a model containing uric acid, hypoxanthine, and lysophosphatidylethanolamine was constructed. Receiver-operating characteristic (ROC) analysis confirmed the prognostic value of these parameters for the diagnosis of minimal/mild endometriosis with a sensitivity of 66.7% and a specificity of 90.0%.

**Conclusions:**

Metabolomics analysis of the eutopic endometrium in endometriosis was effectively characterized by UHPLC-ESI-HRMS-based metabolomics. Our study supports the importance of purine and amino acid metabolites in the pathophysiology of endometriosis and provides potential biomarkers for semi-invasive diagnosis of early-stage endometriosis.

## Background

Endometriosis is a chronic, benign gynaecological disorder characterized by the presence of endometrial cells at extrauterine sites and associated with chronic pain and infertility. This disease is a highly prevalent disease, presenting in 10–15% of reproductive age women and approximately 25 to 50% of infertile women [1, 2]. Endometriosis has a severe impact on socioeconomics and the quality of life of patients [3]. Endometriosis is classified into minimal (I), mild (II), moderate (III) and severe (IV) stages [4]. The incidence of minimal or mild endometriosis is more frequent than advanced endometriosis. Minimal or mild endometriosis is peritoneal or ovarian endometriotic implants and filmy adhesions on the fallopian tubes or ovaries. The presence of early-stage endometriosis is associated with poor oocyte quality, lower fertilization rate and embryonic developmental competence [5, 6]. However, no substantial pelvic anatomical changes have been identified. In addition, atypical symptoms or even no symptoms increase the difficulty of diagnosis in minimal or mild endometriosis, which can be delayed on average by 8 to 11 years [7]. Currently, the sensitivity and specificity of non-invasive diagnostic methods for endometriosis, especially early-stage, are not optimal. The clinical diagnostic indicator cancer antigen 125 (CA125) performs poorly in diagnosing minimal endometriosis, with a sensitivity of 24% [8]. Therefore, it is urgent to explore novel diagnostic biomarkers.

Metabolomics has emerged as a powerful and reliable tool to identify metabolites and biomarkers present in the biological system under a given physiological condition. Metabolites not only represent the final products of biological regulatory processes but also act as communicators between the information-rich genome and the functional phenotype. In the past few years, several studies identified a list of potential diagnostic candidates in peritoneal fluid, blood and urine from endometriosis patients at different stages of disease and menstrual cycle [9, 10]. However, potential biomarkers from the eutopic endometrium remain unknown. Therefore, in the current study, ultra-high-performance liquid chromatography coupled with electrospray ionization high-resolution mass spectrometry (UHPLC-ESI-HRMS) was used to investigate the metabolomic profile of the eutopic endometrium between minimal/mild endometriosis patients and controls. Twelve metabolites were identified as endometriosis-associated biomarkers. The eutopic endometrium metabolomic profile of endometriosis patients was characterized by a significant increase in the concentration of hypoxanthine, L-arginine, L-tyrosine, leucine, lysine, inosine, omega-3 arachidonic acid, guanosine, xanthosine, lysophosphatidylethanolamine and asparagine. In contrast, the concentration of uric acid was decreased. Our study provides potential biomarkers for the semi-invasive diagnose of endometriosis at minimal-mild stages.

## Methods

### Subject selection

Patient recruitment was carried out at the Sixth Hospital of Sun Yat-sen University, and analysis of the endometrium metabolomic profiles was performed at the School of Pharmaceutical Sciences at Sun Yat-sen University. Eutopic endometrium was collected from 68 volunteers (21–38 years old, body mass index less than 30 kg/m^2^) from January 2014 to January 2015 who underwent diagnostic laparoscopy combined with hysteroscopy because of infertility. Clinical diagnosis and classification of subjects were performed through laparoscopic surgery to visually confirm the presence of endometriotic lesions. Surgery was carried out on the third to fifth day after menstrual cessation. All participants had regular menstrual cycles (between 21 and 35 days) without hormonal treatment or use of an intrauterine device in the 3 months before sample collection. Endometrial tissues were obtained via Pipelle biopsy during surgery on the 3rd-5th day after the end of their menstrual bleeding. The severity of endometriosis was determined according to the American Society of Reproductive Medicine revised system [4]. Patients diagnosed with endometrial polyp, endometritis, submucous myoma or hydrosalpinx should be excluded after confirmation with hysteroscopy combined with laparoscopy and further confirmation by histology. Two volunteers diagnosed by hysteroscopy with endometrial polyps were excluded. No other pathologies were detected in the 68 volunteers. Three volunteers were highly suspicious for endometrioma on 2 ultrasounds with more than 3 months interval. The mean sizes of the cysts were 8 mm, 7 mm and 8 mm, which were confirmed during surgery. Clinical information associated with each sample group is summarized in Table 1. After collection, specimens were immediately placed into microtubes and preserved in liquid nitrogen until analysis. This study received the approval of the institutional review board, and all patients gave their written informed consent (approval number: G2012021).

**Table 1:** Characteristics of participants for endometriosis patients and controls

	Endometriosis patients (*n* = 29)	Control group (*n* = 37)	*P*
Age (years)	29.69 ± 3.19	29.74 ± 3.43	0.9543
BMI (kg/m^2^)	21.04 ± 2.08	21.89 ± 3.22	0.2916
AMH (ng/ml)	4.35 ± 2.74	6.4 ± 4.68	0.0661
Uric acid(μmol/l)	271.2 ± 67.38	289.1 ± 63.15	0.3018
The day of sampling	8.667 ± 0.2108	8.45 ± 0.3033	0.5576
The length of menstruation	5.19 ± 0.3209	5 ± 0.3839	0.7044
Endometriosis stage			
I stage	19	N/A	
II stage	10	N/A	
Ovarian endometriomas	3	N/A	

### Sample preparation for metabolomics

Endometrial tissues were obtained from 37 healthy women (Control) and 29 women with endometriosis. Sample preparation was performed according to a previous report with slight modifications [11]. Briefly, 400 μL of 50% chilled methanol was added to 20 mg of tissue sections in tubes containing ceramic beads for homogenization by using a Precellys 24 homogenizer (Bertin, France). The supernatant was transferred into a fresh tube, and 800 μL of chilled 100% acetonitrile was added to precipitate the protein. Samples were centrifuged at 18000×g at 4 °C for 15 min. A total of 500 μL of supernatant was transferred to a fresh tube and dried under vacuum. Samples were re-suspended in 200 μL of 70% acetonitrile for hydrophobic interaction liquid chromatography (HILIC) mode or in 35% acetonitrile for reversed-phase liquid chromatography (RPLC) mode and then centrifuged at 18000×g at 4 °C for 5 min. Finally, 5 μL of supernatant was transferred to a UPLC vial and injected for UHPLC-ESI-HRMS analysis. The quality control (QC) samples comprised 5 μL of each sample, representing a universal set of metabolites for this study. In addition, blank samples were 70% acetonitrile or 35% acetonitrile.

### UHPLC-ESI-HRMS measurements of endometrial tissues

According to our previously reported method [12], chromatography was performed using an Ultimate 3000 HPLC system (Dionex Corporation, Sunnyvale, CA) coupled to a Q Exactive™ benchtop Orbitrap high-resolution mass spectrometer (Thermo Fisher Scientific, San Jose, CA). For the HILIC mode, an Atlantis Silica HILIC 3 μm column (100 mm × 2.1 mm, Waters, Milford, MA, USA), total run time 30 min, was employed. Solvent A was 95% acetonitrile containing 10 mM ammonium formate and 0.1% formic acid, and solvent B was 10 mM ammonium formate and 0.1% formic acid in 50% acetonitrile. The linear gradient used was as follows: holding in 100% A for 0–1 min, increasing to 100% B linearly for 20 min and washing the column for the next 4.9 min, then returning to 100% A until 30 min for column equilibration with a flow rate of 0.3 mL/min. For the RPLC mode, samples were injected onto an Xterra MS C18 5 μm column (100 mm × 2.1 mm, Waters, Milford, MA, USA). The mobile phase consisted of 0.1% formic acid in water (A) and 100% acetonitrile (B). The flow rate was kept at 0.3 mL/min during a 22-min run with the following gradient: 100% A for 2 min to 52% A at 4 min to 30% A at 11 min to 25% A at 14 min and kept at 100% B from 16 min to 17 min and 100% A from 18 min to 22 min. The column temperature was kept at 40 °C. Mass spectrometry was performed with an electrospray ionization source both in positive and negative ionization modes under the following conditions: the spray voltage was 3.5 kV. The capillary and aux gas heater temperature were 300 °C and 350 °C, respectively. Nitrogen was used as sheath gas (40 arbitrary) and auxiliary gas (10 arbitrary). Data were acquired from 80 to 900 mass-to-charge (*m/z*) for mass scanning, and the step collision energy 15, 30, 45 eV was used for MS/MS fragmentation of ions. QC samples were injected intermittently to account for the reproducibility and stability of the UHPLC-ESI-HRMS data [13].

### Data analysis

The mass spectra data were pre-processed by SIEVE 2.2 (Thermo Fisher Scientific, San Jose, CA) to remove the background and generate a multivariate data matrix containing aligned peak areas with matched *m/z* and retention times. Then, SIMCA 13.0 software (Umetrics, Kinnelon, NJ) was applied to find the features that were responsible for the discrimination of the groups. An orthogonal partial least squares discriminant analysis (OPLS-DA) was used to maximize the group discrimination. The candidate markers were selected by examining the S-plot based on the variable importance (VIP) value, which was more than 1.0. The identification of the metabolites was confirmed by comparisons of fragmentation spectra and *m/z* through three main online databases: Metlin (http://metlin.scripps.edu), HMDB (http://www.hmdb.ca/), and mzcloud (https://www.mzcloud.org/) [14, 15]. To assess the strength of association between individual metabolites and minimal/mild endometriosis, a step-wise logistic regression analysis with backward elimination was used to establish a model and filter crucial metabolites. The receiver operating characteristic (ROC) curve was plotted, and the area under the curve (AUC) was calculated. The optimal point on the ROC curve provided the best trade-off between sensitivity and specificity. Statistical testing was carried out by SPSS 19.0 software (IBM Analytics, USA). Data were assessed for normality of distribution using the Shapiro–Wilk test first. Unpaired Student’s t-test or the non-parametric Mann–Whitney U-test was evaluated with a 95% confidence level for statistical analysis between the two groups. False discovery rate (FDR) control was performed by the SAS PROC MULTITEST with the FDR option (SAS Inst, Cary, North Carolina, USA). *P*-values less than 0.05 were considered statistically significant while controlling FDR at 0.05.

## Results

### Characteristics of participants with endometriosis and controls

Clinical information associated with each sample group is summarized in Table 1. A total of 66 volunteers were recruited in this study. Twenty-nine patients had laparoscopically confirmed endometriosis, staged as minimal (*n* = 19) and mild (*n* = 10). Three patients had a laparoscopically documented presence of ovarian endometrioma. All of the endometriomas were histologically confirmed. The mean sizes of all the cysts were less than 1 cm. Age, BMI, menstrual cycle, AMH and uric acid in serum were comparable between the two groups (*P* > 0.05). Both the mean day of sampling and the length of menstruation were not significantly different between the endometriosis patients and the control group. No volunteer had a history of smoking in this study.

### Multivariate statistical analysis of difference between the endometriosis and control groups

**Fig. 1:**
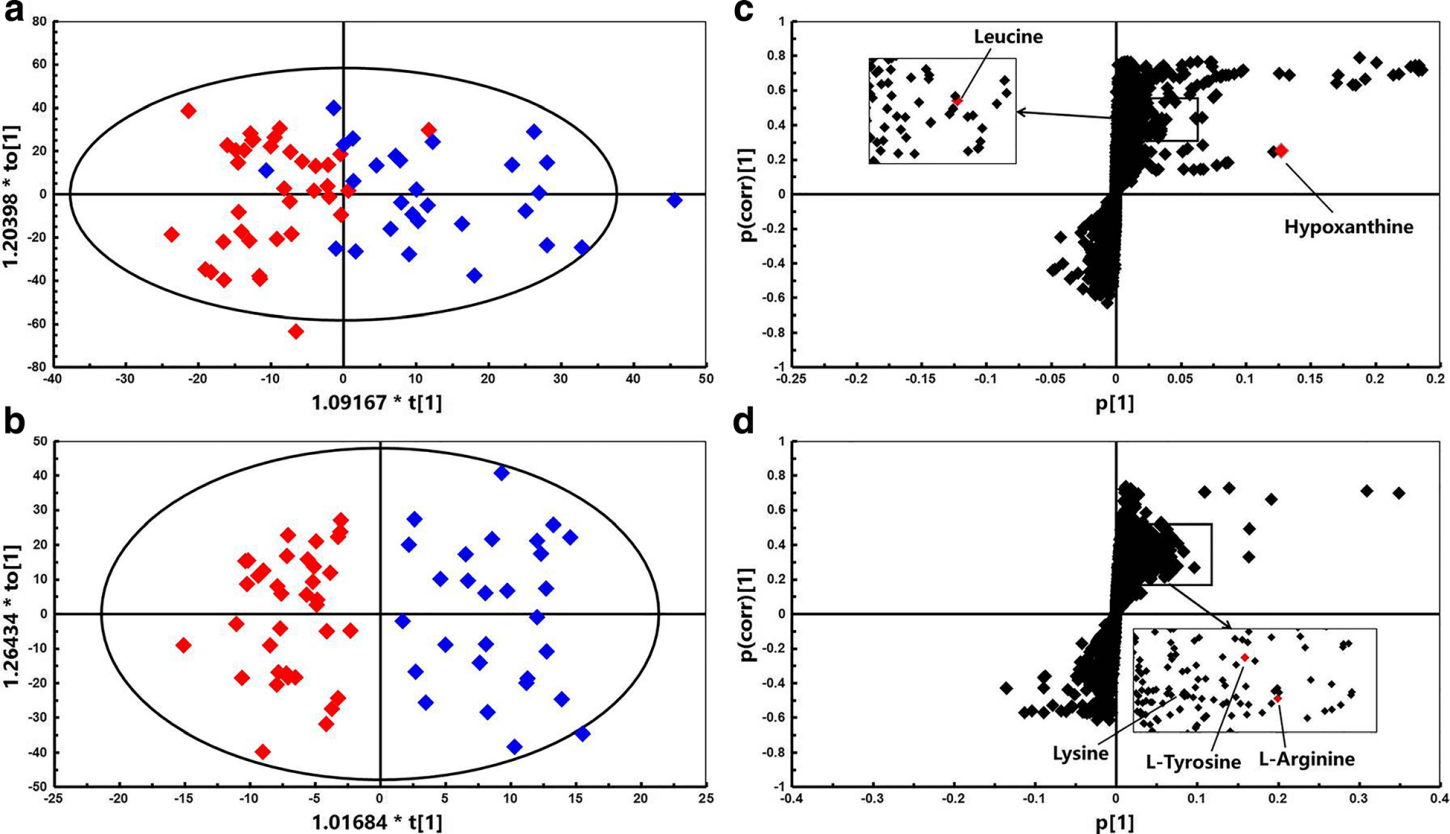
Metabolomic analysis of endometrial tissues from patients with endometriosis (*n* = 29, *blue diamonds*) and healthy controls (*n* = 37, *red diamonds*) under positive ionization mode. Score scatter plots for HILIC (**a**) and RPLC (**b**) modes and OPLS-DA loadings S-plots for HILIC (**c**) and RPLC (**d**) modes. The major ions are labelled in the S-plot

**Fig. 2:**
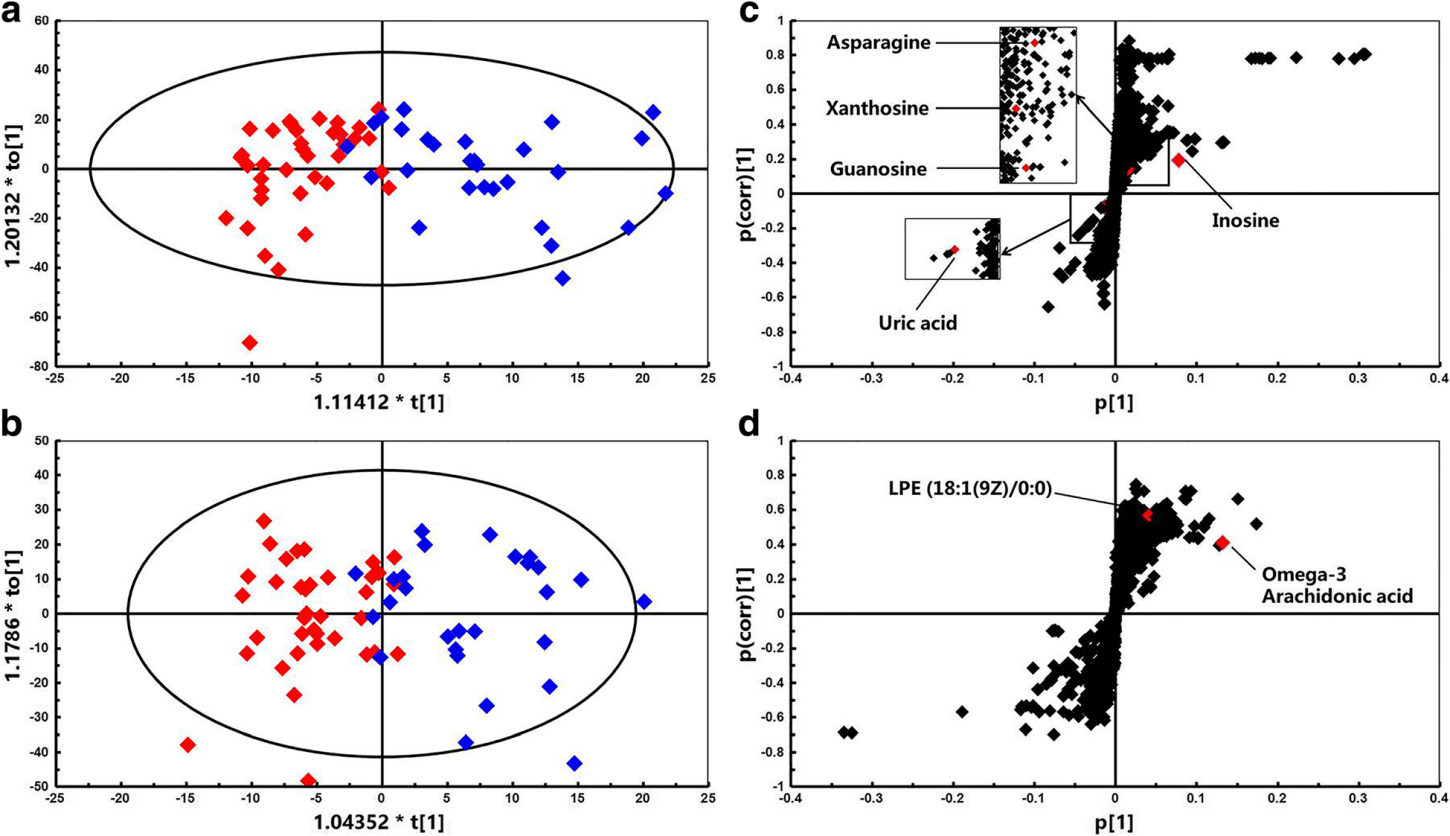
Metabolomic analysis of endometrial tissues from patients with endometriosis (*n* = 29, *blue diamonds*) and healthy controls (*n* = 37, *red diamonds*) under negative ionization mode. Score scatter plots for HILIC (**a**) and RPLC (**b**) modes and OPLS-DA loadings S-plot for HILIC (**c**) and RPLC (**d**) modes. The major ions are labelled in the S-plot

The alignment of all the features in all samples generated a data matrix by SIEVE 2.2 software with an abundance of 5388 features under HILIC mode and 3424 features under RPLC mode. To compare the overall variation of metabolic profiles between the endometriosis patients and healthy controls, a classification model was built by the supervised OPLS-DA, which revealed a clear separation between the two groups (Figs. 1a, b and 2a, b). The model also showed that samples from humans had great individual differences. An OPLS-DA loadings S-plot was performed to highlight significantly different variables in the two groups (Figs. 1c, d and 2c, d). Each point represented a detected ion (variables). The further away from the plot origin an ion point lies, the more the ion contributes to the difference between the two study groups. Therefore, variables plotted at the top or bottom were changed most significantly. Metabolite features of interest were selected by a VIP value > 1.0. With such a strategy, 450 variables from the HILIC mode results and 469 variables from the RPLC mode results were considered to have impact on the model.

### Identification of detected metabolites

**Fig. 3:**
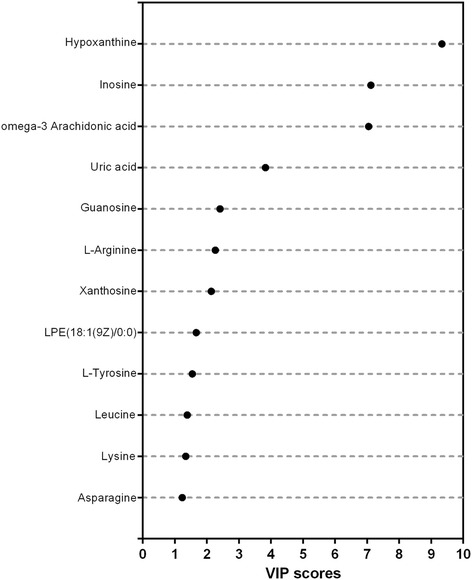
Identified metabolites with increasing contributions to the difference in metabolomic profiles between the two groups based on VIP scores

**Fig. 4:**
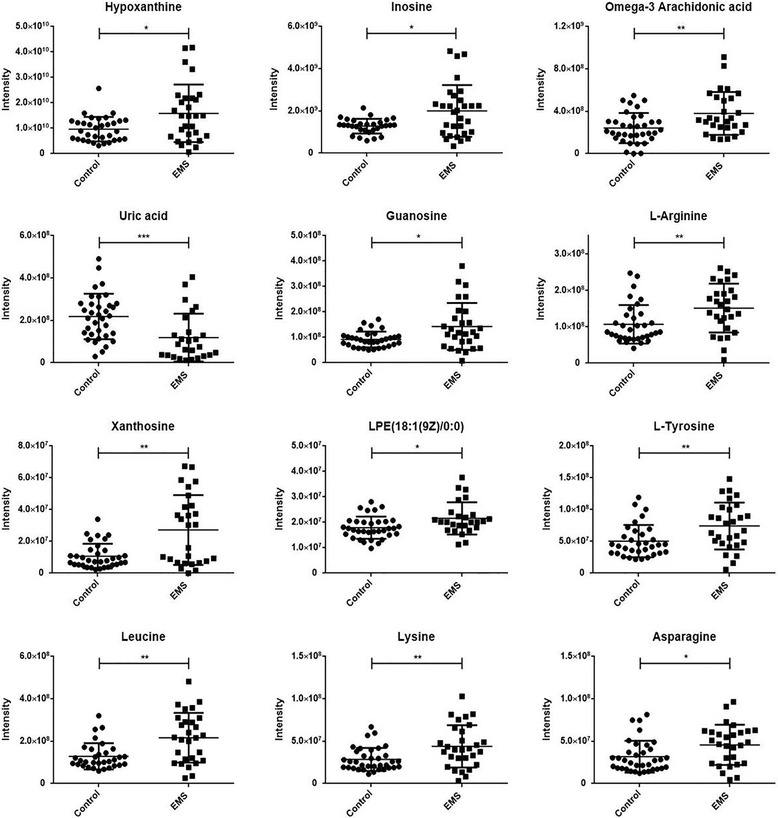
Scatter diagram of 12 selected metabolites. Data are expressed as the mean ± SD. *P < 0.05, **P < 0.01, ***P < 0.001, endometriosis patients (EMS, *n* = 29) vs healthy controls (Control, *n* = 37)

**Fig. 5:**
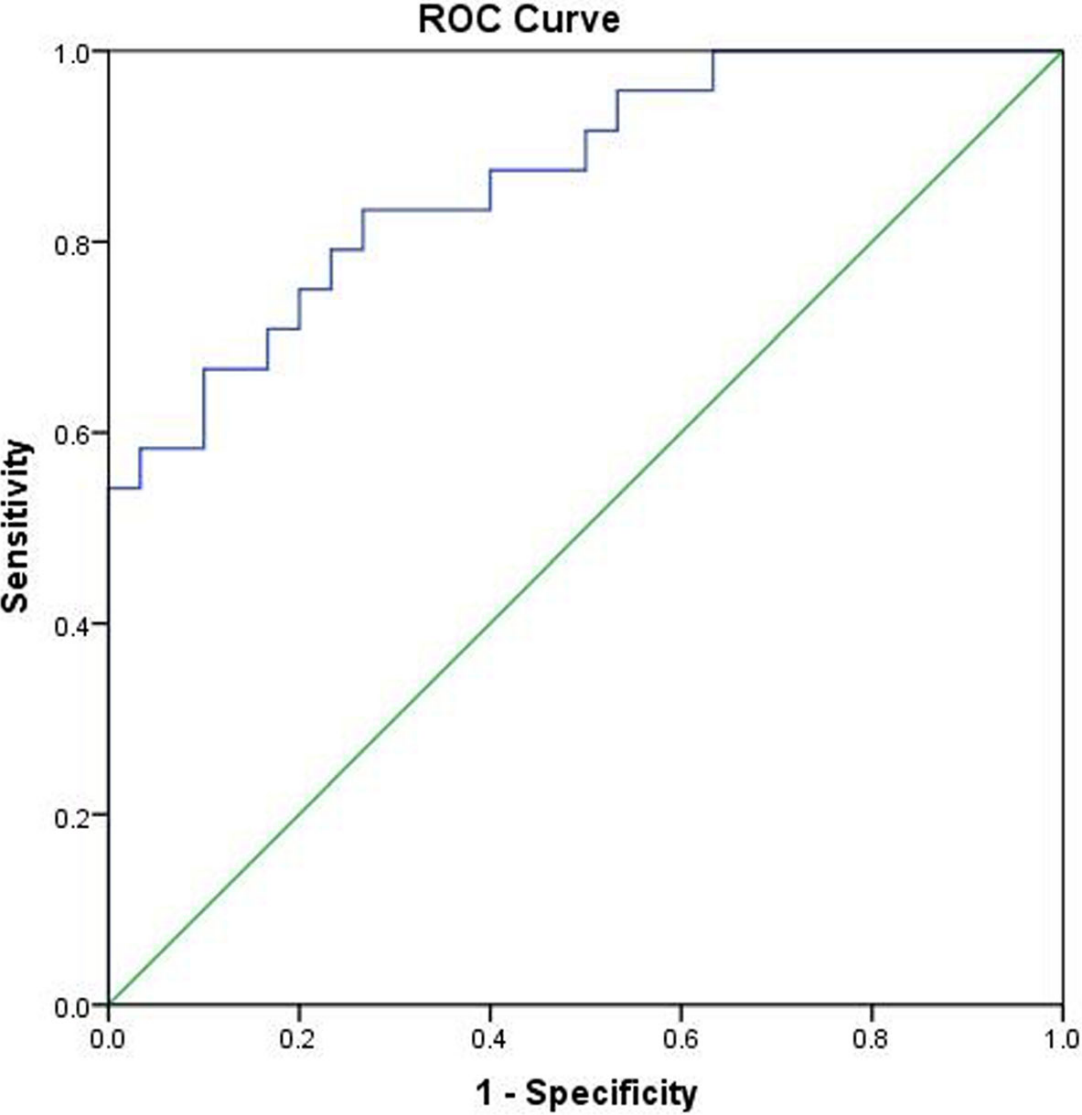
Receiver operating characteristic curves for the model of endometriosis at minimal/mild stages

The *m/z* of the selected variables and their MS/MS fragmentation spectra were used for comparison with compounds annotated in the online databases. Finally, 27 metabolites from positive and negative ionization modes were uniquely identified on the basis of exact mass and retention time. Among them, levels of 12 metabolites corresponding to high variable importance (VIP > 1) (Fig. 3) were different between the endometriosis and control groups (*P* < 0.05). In addition, their detailed information is summarized in Table 2 and labelled in the S-plot (Figs. 1c, d and 2c, d). Obviously, levels of hypoxanthine, L-arginine, L-tyrosine, leucine, lysine, inosine, omega-3 arachidonic acid, guanosine, xanthosine, lysophosphatidylethanolamine and asparagine were higher in the endometriosis group than in the control group, whereas the level of uric acid was higher in the control group (Fig. 4). It is noteworthy that the xanthosine level in the endometriosis group was 2.53-fold higher than that of the control group, while the amount of uric acid was decreased by half, indicating that purine metabolism was disturbed in endometriosis patients. After using step-wise multivariate logistic regression analysis with backward elimination, a model with three predictors was established, including uric acid, hypoxanthine and lysophosphatidylethanolamine, with a sensitivity of 66.7% (95% CI: 0.417–0.875) and a specificity of 90.0% (95% CI: 0.600–1.000). The receiver operating characteristic (ROC) curve shows improved effects of adding separate variables to the model. The apparent AUC of the ROC curve for the model predicting endometriosis at the minimal/mild stages was 0.868 (95% CI: 0.774–0.963) (Fig. 5). The combination of three variables led to a curve with significantly better performance and allows very good discrimination between endometriosis patients at early stages and controls.

**Table 2:** Summary of the data from the 12 features found in positive and negative ionization modes contributing to the discrimination of endometrial tissues between endometriosis patients and healthy controls

*m/z* ^a^	t_R_ (min)^b^	Metabolite	Molecular formula	Adduct	Fold change^c^	*P* value^d^	Adj *P* value^e^
HILIC mode							
131.0462	10.793	Asparagine	C_4_H_8_N_2_O_3_	M-H	1.44	0.013	0.0173
132.1019	8.833	Leucine	C_6_H_13_NO_2_	M + H	1.68	0.002	0.0120
137.0455	4.431	Hypoxanthine	C_5_H_4_N_4_O	M + H	1.64	0.033	0.0360
167.0209	5.283	Uric acid	C_5_H_4_N_4_O_3_	M-H	0.54	0.000	0.0053
267.0737	4.539	Inosine	C_10_H_12_N_4_O_5_	M-H	1.58	0.037	0.0370
282.0844	6.017	Guanosine	C_10_H_13_N_5_O_5_	M-H	1.55	0.023	0.0276
283.0685	4.771	Xanthosine	C_10_H_12_N_4_O_6_	M-H	2.53	0.008	0.0137
RPLC mode							
147.1125	0.696	Lysine	C_6_H_14_N_2_O_2_	M + H	1.54	0.008	0.0137
175.1186	0.703	L-Arginine	C_6_H_14_N_4_O_2_	M + H	1.41	0.004	0.0137
182.0808	1.091	L-Tyrosine	C_9_H_11_NO_3_	M + H	1.47	0.006	0.0137
303.2329	15.245	Omega-3 Arachidonic acid	C_20_H_32_O_2_	M-H	1.57	0.005	0.0137
478.2935	10.770	LPE (18:1(9Z)/0:0)	C_23_H_46_NO_7_P	M-H	1.20	0.013	0.0173

^a^*m/z* is the detected mass to charge ratio from LC-MS/MS runs

^b^Retention time in minutes

^c^The fold change of the endometriosis group vs the control group (a higher ratio indicates a higher level of expression of a compound in the EMS group)

^d^*P* value is the significance level of the difference between the two groups

^e^*P* values were adjusted for false discovery rate correction at the significance level of 5%

*LPE* lysophosphatidylethanolamine, *PC* phosphatidylcholine

## Discussion

In the current study, we applied a UHPLC-ESI-HRMS-based metabolome profiling approach to investigate metabolic changes in the eutopic endometrium samples from endometriosis patients and identified metabolites for early-diagnosed endometriosis. In this regard, 11 metabolites including hypoxanthine, L-arginine, L-tyrosine, leucine, lysine, inosine, omega-3 arachidonic acid, guanosine, xanthosine, lysophosphatidylethanolamine and asparagine were significantly increased in the endometriosis group, whereas the uric acid level was decreased. The global metabolomics and subsequent multivariate analysis clearly distinguished metabolic changes in the endometriosis patients from those in the matched controls. A combination of three predictors (uric acid, hypoxanthine and lysophosphatidylethanolamine) shows a very good potential for use in diagnosing endometriosis at early stages. However, a study with a larger sample size is needed to obtain stronger evidence and avoid wide confidence intervals in the future.

Endometriosis is a disease characterized by the presence of endometrial glands and stroma at ectopic sites. This gynaecological disease occurs in approximately 10% of women of reproductive age, who present symptoms including dyspareunia, dysmenorrhoea, chronic pelvic pain and subfertility [16]. Laparoscopy is the gold standard for the diagnosis of endometriosis. However, laparoscopy is an invasive operation with several limitations, such as surgery-associated risks and financial burden [17]. So far, it has not been able to accurately predict the presence of endometriosis based on non-invasive way. Ultrasound could efficiently detect the presence of ovarian endometriomas, but it is inadequate for the diagnosis of peritoneal endometriosis, deep endometriosis and endometriosis-associated adhesions. CA125 is the most frequently studied biomarker for endometriosis [18]. However, it may be more beneficial for diagnosing advanced stages (III–IV) compared to early stages (I - II) [19]. Hirsch et al. showed that CA 125 performs poorly in diagnosing minimal endometriosis, with a sensitivity of 24% [8]. At present, over 100 potential biomarkers of endometriosis have been reported; however, few markers were useful for the detection of minimal–mild endometriosis [20]. The diagnosis of endometriosis can be delayed, on average, by 8 to 11 years, which leads to significant symptoms [7]. Thus, the cost-effectiveness of endometriosis diagnosis and therapy should be urgently improved.

Increasing evidence shows that metabolomics using easily accessible human biosamples has become an effective tool to explore diagnostic biomarkers and investigate disease progression [21–24]. Metabolomics analysis in endometriosis has been performed in peripheral blood, peritoneal fluid, follicular fluid and urine [25–28]. According to the widely accepted theory of retrograde menstruation, the endometrium is the source of ectopic endometriotic foci. Previous studies showed that the eutopic endometrium contributed to the pathogenesis of endometriosis due to the increase of proliferation, migration and invasion of ectopic endometrium [29–33]. In this study, we did not detect a significant difference in patients’ uric acid level in serum. However, uric acid level was reduced by about half in the eutopic endometrium of patients. The differential expression of uric acid in the serum and endometrium indicated that the eutopic endometrium was more representative, stable and similar to ectopic lesions compared to other samples. In addition, we utilized a semi-invasive way of sampling. The pipelle endometrial biopsy can be used without cervical dilatation in the outpatient department and causes minimal discomfort. Thus, metabolomics analysis via pipelle endometrial biopsy is a viable method to explore molecular markers of endometriosis. All the samples were obtained strictly on the third to fifth day after menstrual cessation because we tried to examine samples in the early follicle phase. Although theoretically we should sample on the same day of the menstrual cycle, each patient’s menstrual period and speed of follicle growth varies. We chose this time to collect samples based on hysteroscopic surgical requirements and patient compliance. Unfortunately, we did not collect enough data on patients with advanced endometriosis to analyse because few patients in stages III-IV in our centre had not been exposed to hormonal drugs within 3 months.

Purine metabolites, including inosine, xanthosine, guanosine and hypoxanthine, were significantly upregulated in the eutopic endometrium, whereas uric acid, as the end product of purine metabolism, was remarkably downregulated. This observation indicates that local purine salvage is potentially impaired. Multiple enzymes participate in the purine metabolism process. Among them, purine nucleoside phosphorylase (PNP) is one of the essential enzymes mediating the generation of uric acid from purines. High levels of expression of this enzyme are postulated to reflect extensive programmed cell death during the implantation process [34, 35]. In addition, pharmacological inhibition of PNP has been demonstrated to be embryo-lethal or teratogenic [34]. Our data indicated that the accumulation of these purine metabolites and decrease in uric acid level in the eutopic endometrium may be due to suppressed PNP expression. A previous study applied parallel gene expression profiling using high-density oligonucleotide microarrays to investigate the regulation of gene expression in the endometrium [36], and reduction of PNP expression was found in endometriosis patients, which supports our hypothesis.

Endometriosis has been known to exhibit similar features of malignancy [37, 38]. Clinical and microscopic examination proved that endometriosis exhibited cancer-like characteristics, demonstrated by uncontrolled growth, cell invasion, neovascularization and apoptosis [39]. Almost all of the amino acids have been reported to be upregulated in carcinoma tissues in previous studies [40]. In cancer cells, a high energy demand leads to the alteration of biochemistry including citric acid cycle dysfunction [41]. Therefore, alternative routes of carbon backbone delivery are required. The increased ectopic endometrium levels of L-arginine, L-tyrosine, leucine, lysine and asparagine observed in the present study might be caused by the alteration of energy metabolism and high turnover of structural protein. These observations are in agreement with a study carried out on serum samples of endometriosis [42] and metabolic alterations observed in oesophageal cancer patients [43, 44].

## Conclusion

Metabolomics provides a powerful approach to explore diagnostic biomarkers by analysing changes in metabolic profiles. Overall, this study is the first to demonstrate a comprehensive analysis of metabolic changes in the eutopic endometrium in endometriosis at early stages. Metabolites involved in purine, amino acid and arachidonic acid metabolic pathways could be potential biomarkers for early diagnosis of endometriosis. These findings provide potential biomarkers for semi-invasive diagnosis of endometriosis at minimal-mild stages in clinical practice. The implications of these individual metabolites in the pathophysiology and analysis of metabolites in all stages of endometriosis have now to be further studied.

## References

[1] Olive DL, Pritts EA (2001). Treatment of endometriosis. N Engl J Med.

[2] Counseller VS, Crenshaw JL (1951). A clinical and surgical review of endometriosis. Am J Obstet Gynecol.

[3] Simoens S, Dunselman G, Dirksen C, Hummelshoj L, Bokor A, Brandes I, Brodszky V, Canis M, Colombo GL, DeLeire T (2012). The burden of endometriosis: costs and quality of life of women with endometriosis and treated in referral centres. Hum Reprod.

[4] Revised American Society for Reproductive Medicine classification of endometriosis (1996). Fertil Steril.

[5] Barnhart K, Dunsmoor-Su R, Coutifaris C (2002). Effect of endometriosis on in vitro fertilization. Fertil Steril.

[6] Bergendal A, Naffah S, Nagy C, Bergqvist A, Sjoblom P, Hillensjo T (1998). Outcome of IVF in patients with endometriosis in comparison with tubal-factor infertility. J Assist Reprod Genet.

[7] Hadfield R, Mardon H, Barlow D, Kennedy S (1996). Delay in the diagnosis of endometriosis: a survey of women from the USA and the UK. Hum Reprod.

[8] Hirsch M, Duffy J, Davis CJ, Nieves Plana M, Khan KS (2016). International collaboration to harmonise O, measures for E: diagnostic accuracy of cancer antigen 125 for endometriosis: a systematic review and meta-analysis. BJOG.

[9] Ahn SH, Singh V, Tayade C (2017). Biomarkers in endometriosis: challenges and opportunities. Fertil Steril.

[10] Marianna S, Alessia P, Susan C, Francesca C, Angela S, Francesca C, Antonella N, Patrizia I, Nicola C, Emilio C (2017). Metabolomic profiling and biochemical evaluation of the follicular fluid of endometriosis patients. Mol BioSyst.

[11] Cheema AK, Pathak R, Zandkarimi F, Kaur P, Alkhalil L, Singh R, Zhong X, Ghosh S, Aykin-Burns N, Hauer-Jensen M (2014). Liver metabolomics reveals increased oxidative stress and fibrogenic potential in gfrp transgenic mice in response to ionizing radiation. J Proteome Res.

[12] Bi H, Krausz KW, Manna SK, Li F, Johnson CH, Gonzalez FJ (2013). Optimization of harvesting, extraction, and analytical protocols for UPLC-ESI-MS-based metabolomic analysis of adherent mammalian cancer cells. Anal Bioanal Chem.

[13] Want EJ, Masson P, Michopoulos F, Wilson ID, Theodoridis G, Plumb RS, Shockcor J, Loftus N, Holmes E, Nicholson JK (2013). Global metabolic profiling of animal and human tissues via UPLC-MS. Nat Protoc.

[14] Yu T, Wang Y, Zhang H, Johnson CH, Jiang Y, Li X, Wu Z, Liu T, Krausz KW, Yu A (2016). Metabolomics reveals mycoplasma contamination interferes with the metabolism of PANC-1 cells. Anal Bioanal Chem.

[15] Zhang H, Jiang Y, Wu J, Zheng C, Ran X, Li D, Huang M, Bi H (2017). Metabolic mapping of Schisandra sphenanthera extract and its active lignans using a metabolomic approach based on ultra high performance liquid chromatography with high-resolution mass spectrometry. J Sep Sci.

[16] Dunselman GA, Vermeulen N, Becker C, Calhaz-Jorge C, D'Hooghe T, De Bie B, Heikinheimo O, Horne AW, Kiesel L, Nap A (2014). ESHRE guideline: management of women with endometriosis. Hum Reprod.

[17] Slack A, Child T, Lindsey I, Kennedy S, Cunningham C, Mortensen N, Koninckx P, McVeigh E (2007). Urological and colorectal complications following surgery for rectovaginal endometriosis. BJOG.

[18] Check JH (2009). CA-125 as a biomarker for malignant transformation of endometriosis. Fertil Steril.

[19] Mol BW, Bayram N, Lijmer JG, Wiegerinck MA, Bongers MY, van der Veen F, Bossuyt PM (1998). The performance of CA-125 measurement in the detection of endometriosis: a meta-analysis. Fertil Steril.

[20] May KE, Conduit-Hulbert SA, Villar J, Kirtley S, Kennedy SH, Becker CM (2010). Peripheral biomarkers of endometriosis: a systematic review. Hum Reprod Update.

[21] Zhang A, Sun H, Wang X (2012). Serum metabolomics as a novel diagnostic approach for disease: a systematic review. Anal Bioanal Chem.

[22] Patel NR, McPhail MJ, Shariff MI, Keun HC, Taylor-Robinson SD (2012). Biofluid metabonomics using (1)H NMR spectroscopy: the road to biomarker discovery in gastroenterology and hepatology. Expert Rev Gastroenterol Hepatol.

[23] Weiss RH, Kim K (2011). Metabolomics in the study of kidney diseases. Nat Rev Nephrol.

[24] Rhee EP, Gerszten RE (2012). Metabolomics and cardiovascular biomarker discovery. Clin Chem.

[25] Vicente-Munoz S, Morcillo I, Puchades-Carrasco L, Paya V, Pellicer A, Pineda-Lucena A (2016). Pathophysiologic processes have an impact on the plasma metabolomic signature of endometriosis patients. Fertil Steril.

[26] Vicente-Munoz S, Morcillo I, Puchades-Carrasco L, Paya V, Pellicer A, Pineda-Lucena A (2015). Nuclear magnetic resonance metabolomic profiling of urine provides a noninvasive alternative to the identification of biomarkers associated with endometriosis. Fertil Steril.

[27] Cordeiro FB, Cataldi TR, Perkel KJ, Do Vale Teixeira da Costa L, Rochetti RC, Stevanato J, Eberlin MN, Zylbersztejn DS, Cedenho AP, Turco EG (2015). Lipidomics analysis of follicular fluid by ESI-MS reveals potential biomarkers for ovarian endometriosis. J Assist Reprod Genet.

[28] Rizner TL (2015). Diagnostic potential of peritoneal fluid biomarkers of endometriosis. Expert Rev Mol Diagn.

[29] Joshi NR, Su RW, Chandramouli GV, Khoo SK, Jeong JW, Young SL, Lessey BA, Fazleabas AT (2015). Altered expression of microRNA-451 in eutopic endometrium of baboons (Papio anubis) with endometriosis. Hum Reprod.

[30] Laudanski P, Charkiewicz R, Tolwinska A, Szamatowicz J, Charkiewicz A, Niklinski J (2015). Profiling of selected MicroRNAs in proliferative Eutopic endometrium of women with ovarian endometriosis. Biomed Res Int.

[31] Goteri G, Altobelli E, Tossetta G, Zizzi A, Avellini C, Licini C, Lorenzi T, Castellucci M, Ciavattini A, Marzioni D (2015). High temperature requirement A1, transforming growth factor beta1, phosphoSmad2 and Ki67 in eutopic and ectopic endometrium of women with endometriosis. Eur J Histochem.

[32] Chelariu-Raicu A, Wilke C, Brand M, Starzinski-Powitz A, Kiesel L, Schuring AN, Gotte M (2016). Syndecan-4 expression is upregulated in endometriosis and contributes to an invasive phenotype. Fertil Steril.

[33] Li Y, An D, Guan YX, Kang S (2017). Aberrant methylation of the E-cadherin gene promoter region in endometrium and ovarian Endometriotic cysts of patients with ovarian endometriosis. Gynecol Obstet Investig.

[34] Witte DP, Wiginton DA, Hutton JJ, Aronow BJ (1991). Coordinate developmental regulation of purine catabolic enzyme expression in gastrointestinal and postimplantation reproductive tracts. J Cell Biol.

[35] Hong L, Mulholland J, Chinsky JM, Knudsen TB, Kellems RE, Glasser SR (1991). Developmental expression of adenosine deaminase during decidualization in the rat uterus. Biol Reprod.

[36] Kao LC, Germeyer A, Tulac S, Lobo S, Yang JP, Taylor RN, Osteen K, Lessey BA, Giudice LC (2003). Expression profiling of endometrium from women with endometriosis reveals candidate genes for disease-based implantation failure and infertility. Endocrinology.

[37] Karanjgaokar VC, Murphy DJ, Samra JS, Mann CH (2009). Malignant transformation of residual endometriosis after hysterectomy: a case series. Fertil Steril.

[38] Lin J, Zhang X, Chen Y (2003). Mutagen sensitivity as a susceptibility marker for endometriosis. Hum Reprod.

[39] Munksgaard PS, Blaakaer J (2011). The association between endometriosis and gynecological cancers and breast cancer: a review of epidemiological data. Gynecol Oncol.

[40] Denkert C, Budczies J, Weichert W, Wohlgemuth G, Scholz M, Kind T, Niesporek S, Noske A, Buckendahl A, Dietel M, Fiehn O (2008). Metabolite profiling of human colon carcinoma--deregulation of TCA cycle and amino acid turnover. Mol Cancer.

[41] Chen JQ, Russo J (2012). Dysregulation of glucose transport, glycolysis, TCA cycle and glutaminolysis by oncogenes and tumor suppressors in cancer cells. Biochim Biophys Acta.

[42] Dutta M, Joshi M, Srivastava S, Lodh I, Chakravarty B, Chaudhury K (2012). A metabonomics approach as a means for identification of potential biomarkers for early diagnosis of endometriosis. Mol BioSyst.

[43] Zhang J, Liu L, Wei S, Nagana Gowda GA, Hammoud Z, Kesler KA, Raftery D (2011). Metabolomics study of esophageal adenocarcinoma. J Thorac Cardiovasc Surg.

[44] Zhang J, Bowers J, Liu L, Wei S, Gowda GA, Hammoud Z, Raftery D (2012). Esophageal cancer metabolite biomarkers detected by LC-MS and NMR methods. PLoS One.

