# Correction to: Endometrium metabolomic profiling reveals potential biomarkers for diagnosis of endometriosis at minimal-mild stages

**DOI:** 10.1186/s12958-019-0532-5

**Published:** 2019-11-06

**Authors:** Jingjie Li, Lihuan Guan, Huizhen Zhang, Yue Gao, Jiahong Sun, Xiao Gong, Dongshun Li, Pan Chen, Xiaoyan Liang, Min Huang, Huichang Bi

**Affiliations:** 1 Center of Reproductive Medicine, the Sixth Affiliated Hospital, Sun Yat-sen University, Guangzhou, China; 2 School of Pharmaceutical Sciences in Sun Yat-sen University, 132# Waihuandong Road, Guangzhou, University City, Guangzhou, 510006 People’s Republic of China; 3 Pharmacy Department, the First Affiliated Hospital, Sun Yat-sen University, Guangzhou, China; 4 School of Public Health, Guangdong Pharmaceutical University, Guangzhou, China

**Correction to: Reprod Biol Endocrinol (2018) 16:42**

**https://doi.org/10.1186/s12958-018-0360-z**

The authors regret that the incorrect identification of “omega-3 arachidonic acid” published in the original manuscript [1]. This classification is incorrect as arachidonic acid is an omega-6 polyunsaturated fatty acid. From a mechanistic standpoint, this mistake might have significance, as arachidonic acid is hypothesized to play a role in inflammatory processes. We have carefully double-checked our original data and compared the fragmentation spectra and m/z referring to Metlin Database again. We are sure that the metabolite should be arachidonic acid, instead of omega-3 arachidonic acid. Given that this misclassification is made in several places within the article’s abstract and text, a correction is warranted.

Accordingly, the name “omega-3 arachidonic acid” mentioned in the following parts should be corrected as “arachidonic acid”:

- In the results section of Abstract: “The eutopic endometrium metabolomic profile of the endometriosis patients was characterized by a significant increase in the concentration of hypoxanthine, L-arginine, L-tyrosine, leucine, lysine, inosine, omega-3 arachidonic acid, guanosine……”.
- In the last paragraph of Introduction: “The eutopic endometrium metabolomic profile of endometriosis patients was characterized by a significant increase in the concentration of hypoxanthine, L-arginine, L-tyrosine, leucine, lysine, inosine, omega-3 arachidonic acid, guanosine……”.
- In the first paragraph of the section with the subheading “Identification of detected metabolites” of Results: “Obviously, levels of hypoxanthine, L-arginine, L-tyrosine, leucine, lysine, inosine, omega-3 arachidonic acid, guanosine…..”
- In the first paragraph of Discussion: “In this regard, 11 metabolites including hypoxanthine, L-arginine, L-tyrosine, leucine, lysine, inosine, omega-3 arachidonic acid, guanosine……”
- Figure 2D, “Omega-3 Arachidonic acid”
- In the third row of Figure 3, “omega-3 arachidonic acid”
- In the third graph in the first row of Figure 4, “Omega-3 Arachidonic acid”
- In the 4th row of RPLC mode in Table 2, “Omega-3 Arachidonic acid”

The authors would like to apologize for any inconvenience caused.

## References

[1] Li J (2018). Endometrium metabolomic profiling reveals potential biomarkers for diagnosis of endometriosis at minimal-mild stages. Reprod Biol Endocrinol.

